# Complete mitochondrial genome sequence of the Himalayan Griffon, *Gyps himalayensis* (Accipitriformes: Accipitridae): Sequence, structure, and phylogenetic analyses

**DOI:** 10.1002/ece3.5433

**Published:** 2019-07-09

**Authors:** Lichun Jiang, Liqing Peng, Min Tang, Zhangqiang You, Min Zhang, Andrea West, Qiping Ruan, Wei Chen, Juha Merilä

**Affiliations:** ^1^ Key Laboratory for Molecular Biology and Biopharmaceutics, School of Life Science and Technology Mianyang Normal University Mianyang Sichuan China; ^2^ Ecological Security and Protection Key Laboratory of Sichuan Province Mianyang Normal University Mianyang Sichuan China; ^3^ Centre for Integrative Ecology, School of Life and Environmental Sciences Deakin University Geelong Vic Australia; ^4^ Ecological Genetics Research Unit, Organismal and Evolutionary Biology Research Programme, Faculty Biological & Environmental Sciences University of Helsinki Helsinki Finland

**Keywords:** Accipitridae, genome organization, *Gyps himalayensis*, mitochondrial genome, phylogenetic status

## Abstract

This is the first study to describe the mitochondrial genome of the Himalayan Griffon, *Gyps himalayensis*, which is an Old World vulture belonging to the family Accipitridae and occurring along the Himalayas and the adjoining Tibetan Plateau. Its mitogenome is a closed circular molecule 17,381 bp in size containing 13 protein‐coding genes, 22 tRNA coding genes, two rRNA‐coding genes, a control region (CR), and an extra pseudo‐control region (CCR) that are conserved in most Accipitridae mitogenomes. The overall base composition of the *G. himalayensis* mitogenome is 24.55% A, 29.49% T, 31.59% C, and 14.37% G, which is typical for bird mitochondrial genomes. The alignment of the Accipitridae species control regions showed high levels of genetic variation and abundant AT content. At the 5′ end of the domain I region, a long continuous poly‐C sequence was found. Two tandem repeats were found in the pseudo‐control regions. Phylogenetic analysis with Bayesian inference and maximum likelihood based on 13 protein‐coding genes indicated that the relationships at the family level were (Falconidae + (Cathartidae + (Sagittariidae + (Accipitridae + Pandionidae))). In the Accipitridae clade, *G. himalayensis* is more closely related to *Aegypius monachus* than to *Spilornis cheela*. The complete mitogenome of *G. himalayensis* provides a potentially useful resource for further exploration of the taxonomic status and phylogenetic history of *Gyps* species.

## 1 INTRODUCTION

The Himalayan vulture (*Gyps himalayensis*) from the family Accipitridae has a wide distribution in Southeast Asia. In China it is found along the Himalayas and the adjoining Tibetan Plateau during the breeding season, and from Yunnan province during the winter season (BirdLife International, 2016; Lu, Ke, & Zeng, 2009). It inhabits mountainous areas at altitudes ranging from 1,200 to 6,000 m (Ferguson‐Lees & Christie, 2001). The species’ population trend is currently stable, but future declines are possible because vultures feed on livestock carcasses containing diclofenac, a nonsteroidal anti‐inflammatory pharmaceutical known to cause reproductive failures in birds (Das, Cuthbert, Jakati, & Prakash, 2011; Oaks et al., 2004; Shultz et al., 2004; Swan et al., 2006).

There are currently eight recognized species in the genus *Gyps* including *G. bengalensis*, *G. indicus*, *G. tenuirostris,* and *G. himalayensis* from Asia, *G. africanus*, *G. coprotheres,* and *G. rueppellii* from Africa, and *G. fulvus* from Europe, Africa, and Asia (Ferguson‐Lees & Christie, 2001; Pain et al., 2003). So far, the complete mitochondrial genomes of all species from this genus have not been amplified. In addition, the phylogenetic position of *Gyps* and related genus species has not been fully solved. Phylogenetic relationships in the family Accipitridae have traditionally been proven to be difficult to resolve based on morphological traits (Griffiths, 1994; Jollie, 1976). Due to its simple genomic organization, high rates of evolution and maternal inheritance mode, complete mitochondrial genomes have been proposed to show potential in resolving evolutionary history and taxonomic status (Arshad, Gonzalez, El‐Sayed, Osborne & Wink,^2009^; Boore, 2006; Dowton, Castro, & Austin, 2002; Gibb, Kardailsky, Kimball, Braun, & Penny, 2007; Kan, Yang, et al., 2010; Ren et al., 2014). In the past 10 years, a number of studies performed using mitochondrial genome sequences to clarify avian phylogeny (Kan, Li, et al., 2010; Li, Liu, Zhou, & Gu, 2015; Li, Liu, et al., 2015; Song et al., 2015; Zhang et al., 2012).

The systematic taxonomic status among Falconiform taxa is rather complex and controversial (Jiang et al., 2015; Liu, Li, Du, & Liu, 2017). The family Accipitridae (hawks, eagles, and kites), with other four families (Pandionidae, Sagittariidae, Cathartidae, and Falconidae), was traditionally classified within the order Falconiformes (Cracraft, 1981; Haring, Kruckenhauser, Gamauf, Riesing, & Pinsker, 2001; Livezey & Zusi, 2007). However, according to more detailed morphological studies of several families, accipitrids and falconids are not closely in evolutionary relationship (Haring et al., 2001; Jollie, 1976). Based on his point of view, Falconiformes is polyphyletic, including New World vultures (Cathartidae), and the result is supported by studies of some behavioral traits and molecular data (Wink & Sauer‐Gürth, 2004). Although the taxonomy of order Falconiformes is supported morphologically (Livezey & Zusi, 2001; Mayr & Clarke, 2003), recent studies have shown that they do not support close phylogenetic relationships between the Falconidae and other families included in the order Falconiformes (Burleigh, Kimball, & Braun, 2015; Hackett et al., 2008; Jarvis et al., 2014; Mahmood, Mclenachan, Gibb, & Penny, 2014). According to the viewpoint of Hackett et al. (2008) and the South American Classification Committee, the Falconiformes is only included in the family Falconidae, and the remaining four families are placed in the order Accipitriformes. For a long time, the majority view has been to include accipitrids with the falcons in the Falconiformes, but some authorities have begun to recognize them as a separate group, Accipitriformes (Chesser et al., 2010). Additionally, a recent DNA study indicated that falcons not closely to the accipitrids, accordingly some authors propose that it should not be a part of Falconiformes (Chesser et al., 2012). Instead, it should be placed within Accipitriformes, and include most of the diurnal birds of prey: hawks, eagles, vultures, and many others (Chesser et al., 2012; Liu et al., 2017). However, its phylogenetic position of *Sagittarius serpentarius* is still confusing, and a lot of controversy about its validity is ongoing (Ferguson‐Lees & Christie, 2001). Finally, some authors propose that *S. serpentarius* become an independent family, Sagittariidae (Wink & Sauer‐Gürth, 2004).

In this study, the complete mitochondrial genome of *G. himalayensis* was sequenced and reported for the first time. Based on new data generated from *G. himalayensis* and obtaining complete mitogenome sequence data from GenBank, we tried to elucidate: (a) sequences, features, and structures of the mitogenomes of *G. himalayensis*, (b) taxonomic status phylogenetic relationships of *G. himalayensis* and related genus species, and (c) the phylogenetic relationships Falconidae, Accipitridae, Pandionidae, Sagittariidae, and Cathartidae.

## 2 MATERIALS AND METHODS

### 2.1 Sample collection and genomic DNA extraction

**Figure 1:**
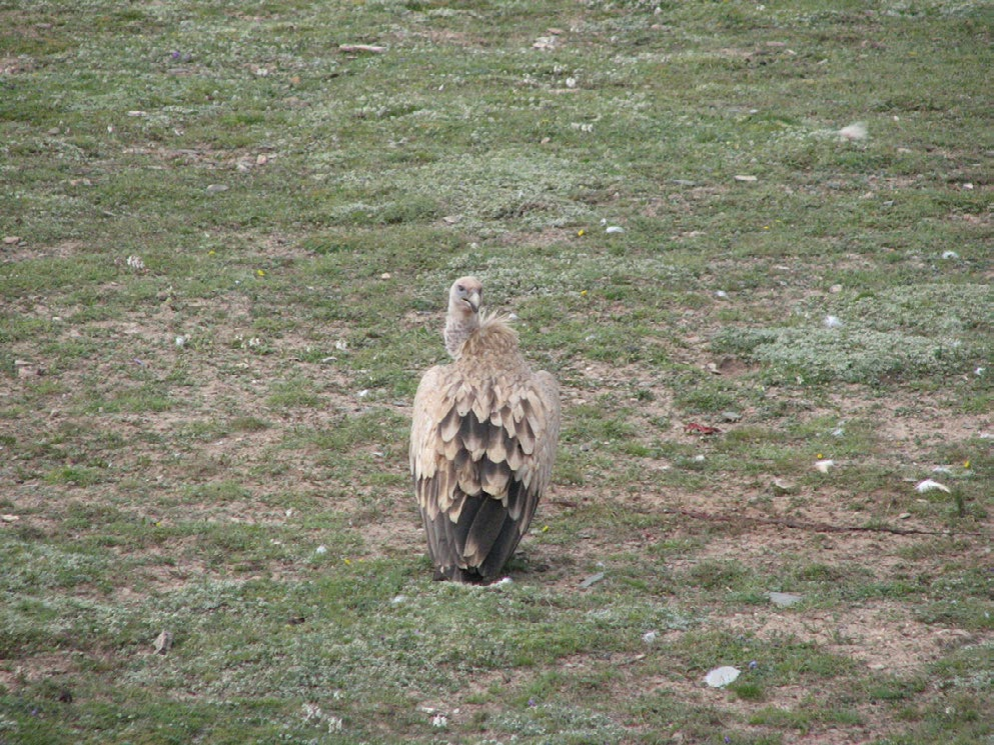
The Himalayan Griffon, *Gyps himalayensis;* photograph taken by Wei Chen

**Figure 2:**
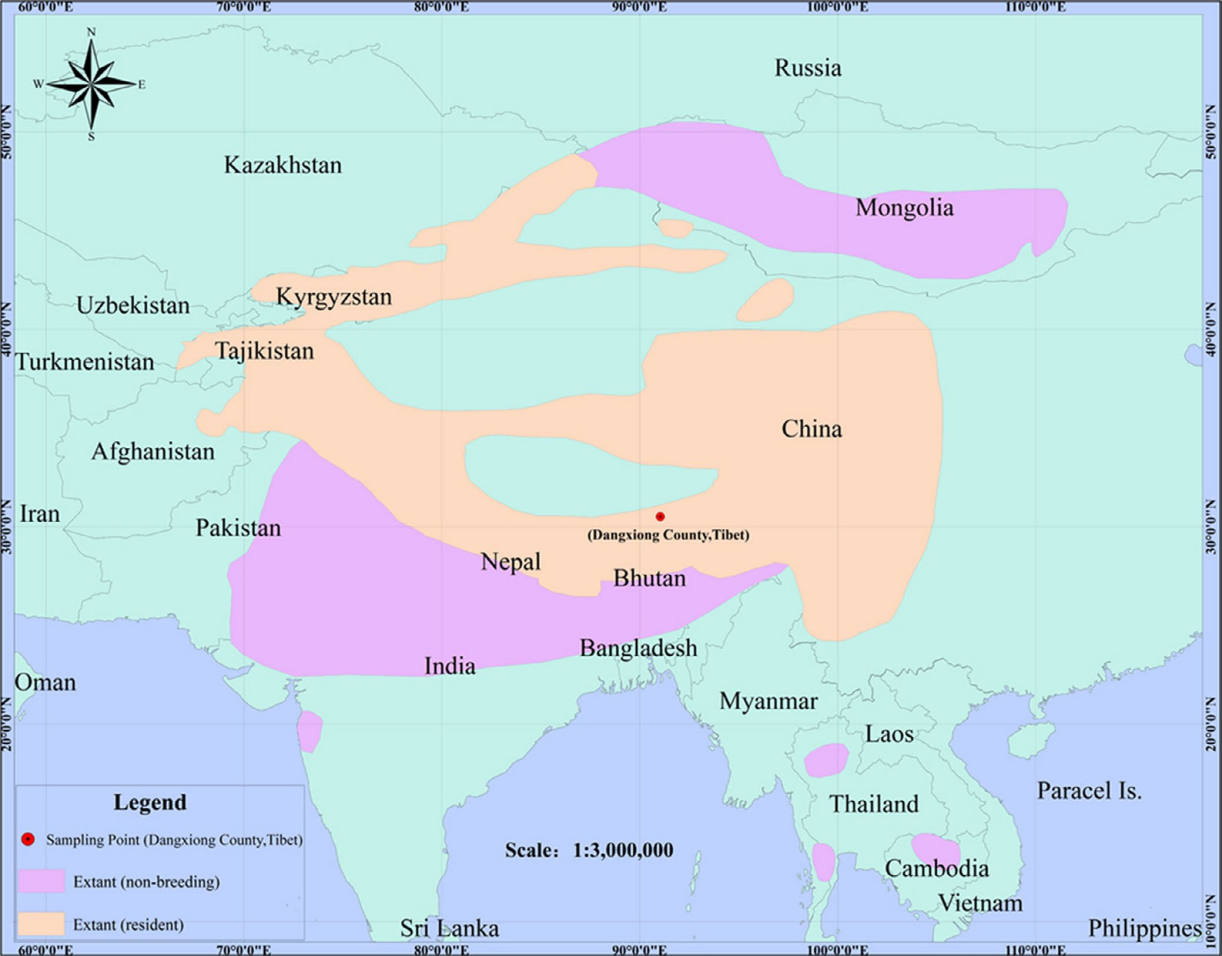
Species distribution map of the Himalayan Griffon, *Gyps himalayensis*

Blood of the Himalayan Griffon, *G. himalayensis* (Figure 1), was sampled from an adult vulture captured in the Dangxiong County (91°5′54.50″E, 30°28′50.79″N, 4,300 m.a.s.l), the Tibet Autonomous Region, China, in July 2016. The distribution of this species is shown in Figure 2. Five milliliters of blood was sampled from the brachial vein using heparinized syringes and stored in liquid nitrogen in field and stored at −80°C in the laboratory. Total genomic DNA was extracted from blood tissue using Phenol: Chloroform: Isoamyl alcohol method (Sambrook & Russell, 2001). After a quality inspection, DNA was purified on 0.8% agarose gel. After measuring DNA concentration using a spectrophotometer, it was used for the PCR amplification.

### 2.2 Mitochondrial DNA amplification and sequencing

The complete mitochondrial genome was amplified in 13 overlapping segments by PCR with Taq DNA Polymerase (TaKaRa), using 20 ng of genomic DNA from the sample as a template. Complete mtDNA was amplified as concatenated sequences using selectively amplified mtDNA template and 11 primer pairs from literature (Jiang, Wang, Peng, Peng, & Zou, 2014; Sorenson; , Ast, Dimcheff, Yuri, & Mindell, 1999; Zhao et al., 2012). Partial PCR primers were designed based on the conserved regions of the related species, *Aegypius monachus* (NC_022957), *Buteo buteo* (NC_003128), and *Spilornis cheela* (NC_015887).The PCR amplifications were conducted in 25 μl reactions containing 1 × PCR Buffer, 200 nM of each primer, 400 μM dNTP, 1 μl template (as per kit), and 1 U LA‐Taq or Taq DNA Polymerase (TaKaRa). The PCR protocol was as follows: an initial denaturation at 95°C for 3 min, followed by 30 amplification cycles with a S1000TM thermal cycler at 94°C for 30 s, 53–65°C for 30 s, and 72°C for 80–180 s, continued with a final extension at 72°C for 10 min. The PCR products were preserved at 12°C after the last cycle. Each amplicon was then purified using the Omega Cycle‐Pure Kit (Omega Bio‐Tek) and subjected to automated sequencing using an ABI 3730 sequencer, either directly or following sub‐cloning into the pUC19 DNA vector (TaKaRa). To ensure high accuracy, each amplicon was sequenced twice independently with all the PCR primers.

### 2.3 Sequence assembly, annotation, and analysis of the mitochondrial genome

The complete mitochondrial genome sequence of the Himalayan Griffon, *G. himalayensis,* was assembled using the SeqMan module of the Lasergene 8.0 software (DNASTAR; Burland, 2000). Protein‐coding genes (PCGs) were identified through sequence comparisons with the known sequences of other birds, using CLUSTAL W program. Transfer RNA (tRNA) genes and their secondary structures were identified using tRNAscan‐SE 1.21 (http://lowelab.ucsc.edu/tRNAScan-SE; Lowe & Eddy, 1997) or by their proposed secondary structures (Kumazawa & Nishida, 1993) and anticodons. Ribosomal RNA genes (rRNAs) were identified by NCBI Internet BLAST search and comparisons. Codon usage and nucleotide composition statistics were computed using MEGA 6.06 (Tamura, Stecher, Peterson, Filipski, & Kumar, 2013).Composition skew analysis was carried out with the formulas AT‐skew = [A–T]/[A + T] and GC‐skew=[G–C]/[G + C], respectively (Perna & Kocher, 1995). The complete mitogenome of *G. himalayensis* was deposited in GenBank under the accession number KY594709.

### 2.4 Phylogenetic analysis

The sequence data were initially compared using program ClustalX 1.83 (Thompson, Gibson, Plewniak, Jeanmougin, & Higgins, 1997) with default parameters. The mitogenome of *G.himalayensis* was analyzed in a phylogenetic tree using a total of 32 other order Falconiformes mitogenomes retrieved from NCBI GenBank (Table S1). The concatenated sequences of the 13 PCGs of complete mitochondrial genomes were used. On the basis of the best‐fit model predicted by jModelTestv.0.1.1, the optimal nucleotide substitution model was selected using jModeltest v.0.1.1 (Posada, 2008) and the Akaike information criterion (Posada & Buckley, 2004). Maximum likelihood (ML) analysis was carried out with PhyML v.3.0 (Guindon & Gascuel, 2003). The confidence level (Felsenstein, 1985) at each branch was evaluated by allowing four substitution rate categories and performing 1,000 bootstrap replicates. BI analysis was performed with MrBayes v.3.1.2 (Huelsenback & Ronquist, 2001). The best‐fitting model of TVM+I+G (nst = 6; rates = gamma) was selected for subsequent Bayesian phylogenetic analyses. Bayesian posterior probabilities were estimated with the Markov Chain Monte Carlo sampling. The analysis was initiated with randomly generated trees and ran for 2 × 10^7^ generations from which a total of 2 × 10^5^ trees were sampled at the intervals of every 100 generations, and among the sampled trees, the top 25% were used as burn‐in and discarded. Tracer v.1.4 (Rambaut & Drummond, 2007) was used to check that sampled values of log likelihood, plotted against generation time, reached stationarity. Finally, a majority rule consensus tree was generated and posterior probabilities were computed from the remaining postburn in trees.

## 3 RESULTS AND DISCUSSION

### 3.1 Genome content and organization

**Figure 3:**
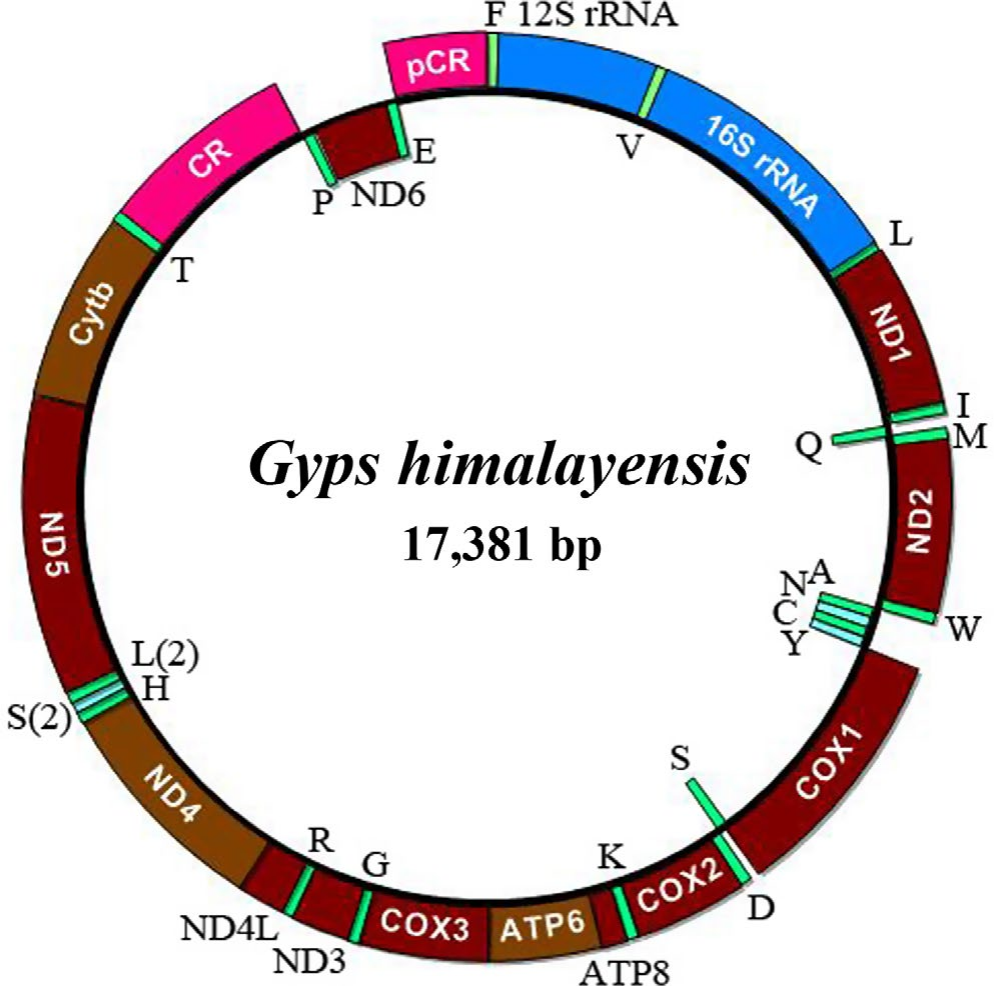
Complete mitochondrial genome organization and gene arrangement of *Gyps himalayensis*. Genes coded on the H‐strand are indicated in the outer ring, while the genes coded on the L‐strand are indicated in the inner ring. Genes are abbreviated as follows: ATP6 and ATP8 (subunits 6 and 8 of ATPase), COXI‐COXIII (cytochrome c oxidase subunits 1–3), Cytb (cytochrome b), ND1‐ND6 and ND4L (NADH dehydrogenase subunits 1–6 and 4L), 12S rRNA and 16S rRNA (ribosomal RNA of 12S and 16S), CR (control region); pCR (pseudo‐coding region). One‐letter amino acid abbreviations were used to label the corresponding tRNA genes

The complete mitogenome of *G. himalayensis* is a closed circular molecule 17,381 bp in length, similar to the other raptors (Li, Liu, et al., 2015; Li, Liu, et al., 2015; Qin, Guan, Shi, Hou, & Qin, 2013). It contains the typical set of 37 genes present in vertebrate mitogenomes, including 13 PCGs, 22 tRNA genes, two genes for ribosomal RNA subunits (12 s rRNA and 16 s rRNA), a control region (CR), and an extra pseudo‐control region (CCR; Table 1, Figure 3). ND6 and eight tRNAs are transcribed from the light strand (L), however the other 12 PCGs, 14 tRNAs, two rRNAs, and two noncoding region (CR, CCR) genes are located on the heavy (H) strand. The gene arrangement pattern of the *G. himalayensis* mitochondrial genome is identical to that in genera *Aegypius*, *Buteo,* and *Falco*, all which have the remnant CCR gene order, but differ from the standard bird gene order (Barker, Benesh, Vandergon, & Lanyon, 2012; Gibb et al., 2007; Li, Huang, et al., 2015). The *G. himalayensis* mitogenome nucleotide composition is biased toward A and T (56.14%: A = 224.55%, T = 29.49%, G = 14.37% and C = 31.59%), which is similar to that seen in the other *Gyps* mitogenomes (52.71%–57.65%; Table S1; Haring et al., 2001). The A + T content of the control region was clearly higher than that of PCGs, tRNAs, and rRNAs (Table S1).

**Table 1:** Characteristics of the mitochondrial genome of *Gyps himalayensis*

Gene	Position		Sizes	Codon		Intergenic nucleotide^b^	Strand^c^	A + T%
	From	To	Nucleotide (bp)	Start	Stop^a^			
tRNA‐Phe	1	70	70				H	48.6
12S ribosomal RNA	71	1,055	985			−19	H	50.9
tRNA‐Val	1,037	1,108	72			−8	H	55.6
16S ribosomal RNA	1,101	2,730	1,630			−20	H	53.4
tRNA‐Leu	2,711	2,784	74			9	H	47.3
ND1	2,794	3,771	978	ATG	AGG	−2	H	53.2
tRNA‐Ile	3,770	3,841	72			13	L	56.9
tRNA‐Gln	3,855	3,925	71			−1	H	66.2
tRNA‐Met	3,925	3,993	69				H	49.3
ND2	3,994	5,038	1,045	ATG	T‐‐		H	51.9
tRNA‐Trp	5,039	5,110	72			1	H	62.5
tRNA‐Ala	5,112	5,180	69			2	L	56.5
tRNA‐Asn	5,183	5,255	73			2	L	47.9
tRNA‐Cys	5,258	5,324	67			−1	L	49.3
tRNA‐Tyr	5,324	5,394	71			1	L	54.9
COXI	5,396	6,946	1,551	GTG	AGG	−9	H	52.5
tRNA‐Ser	6,938	7,011	74			4	L	52.7
tRNA‐Asp	7,016	7,084	69			2	H	59.4
COXII	7,087	7,770	684	ATG	TAA		H	53.2
tRNA‐Lys	7,772	7,843	72			1	H	54.2
ATP8	7,844	8,008	165	ATG	TAA	−10	H	52.1
ATP6	7,999	8,682	684	ATG	TAA	−1	H	54.5
COXIII	8,682	9,464	783	ATG	TAA	1	H	52.6
tRNA‐Gly	9,466	9,534	69				H	66.7
ND3	9,535	9,885	351	ATT	TAA	4	H	56.1
tRNA‐Arg	9,890	9,958	69			1	H	60.9
ND4L	9,960	10,256	297	ATG	TAA	−7	H	54.2
ND4	10,250	11,626	1,377	ATG	TAA		H	51.2
tRNA‐His	11,627	11,696	70			1	H	65.7
tRNA‐Ser	11,698	11,762	65				H	52.7
tRNA‐Leu	11,763	11,833	71				H	62.0
ND5	11,834	13,648	1,815	ATG	TAA	12	H	54.3
Cytb	13,661	14,803	1,143	ATG	TAA	2	H	52.1
tRNA‐Thr	14,806	14,873	68				H	64.7
Control region	14,874	16,075	1,202			8	H	56.7
tRNA‐Pro	16,084	16,153	70			22	L	62.9
ND6	16,176	16,694	519	ATT	TAG	−3	L	50.1
tRNA‐Glu	16,692	16,762	71				L	62.0
Pseudo‐control region	16,763	17,381	619				H	69.0

a T—represents incomplete stop codons.

b Intergenic bp indicates gap nucleotides (positive value) or overlapped nucleotides (negative value) between two adjacent genes.

c H and L indicate genes transcribed on the heavy and light strands, respectively.

Mitochondrial genes overlapped by a total of 151 bp in 12 different locations from 1 to 70 bp, and the longest overlap (70 bp) existed between genes tRNA‐Glu and pseudo‐control region. The shortest overlap (1bp) existed in ATP6‐COXIII, tRNA‐Cys‐tRNA‐Tyr, and tRNA‐Gln‐tRNA‐Met genes. In addition, some protein‐coding genes shared 1–20 nucleotides in common with adjacent tRNA genes. This is a common feature of mitochondrial DNA which is very compact and economical (Curole & Kocher, 1999). Furthermore, 17 intergenic spacers were present in the *G. himalayensis* mitogenome and involved a total of 85 bp. The longest spacer sequence was 22 nucleotides long and located between ND6 and tRNA‐Pro (Table 1).

AT/CG skews were essentially similar in the mitochondrial protein‐coding genes (PCG), 2 rRNA genes, CR and the mitogenomes of other Falconiformes birds, (e.g. *A. monachus*, *F. peregrinus,* and *Micrastur gilvicollis*), except for a few genes (ND3, ND4L and CR; Table 2). Moreover, *G. himalayensis* and *A. monachus* share negative AT‐skew in ND4L and CR genes, whereas *F. peregrinus* and *M. gilvicollis* have positive AT‐skew (Table 2). For ND3 and COX3 genes, *G. himalayensis* has negative AT‐skew, while the other three species have positive AT‐skews. Apparent positive AT‐skew and negative GC‐skew biases were identified for all 11 PCGs (except for ND3 and ND4L) encoded by the H‐strand, whereas the reverse was detected in ND6 encoded by the L‐strand, which has been observed also in previous studies (Table 2; Jiang et al., 2015; Liu et al., 2017).

**Table 2:** AT/CG skews in the mitochondrial protein‐coding genes (PCG), 2 rRNA genes, CR and the entire mitogenomes of Falconiformes birds, *Gyps himalayensis*, *Aegypius monachus*, *Falco peregrinus,* and *Micrastur gilvicollis*

Gene	AT‐skew = A − T/A + T				GC‐skew = G − C/G + C			
	*G. himalayensis*	*A. monachus*	*F. peregrinus*	*M. gilvicollis*	*G. himalayensis*	*A. monachus*	*F. peregrinus*	*M. gilvicollis*
ND1	0.000	0.006	0.023	0.016	−0.410	−0.417	−0.464	−0.446
ND2	0.162	0.144	0.126	0.153	−0.555	−0.549	−0.564	−0.558
ND3	**−0.015**	**0.032**	**0.095**	**0.000**	−0.455	−0.509	−0.516	−0.421
ND4	0.110	0.110	0.150	0.143	−0.545	−0.564	−0.570	−0.569
ND4L	**−0.056**	**−0.012**	**0.031**	**0.051**	−0.397	−0.467	−0.456	−0.447
ND5	0.145	0.152	0.206	0.174	−0.477	−0.508	−0.502	−0.524
ND6	−0.477	−0.496	−0.594	−0.591	0.552	0.580	0.595	0.624
COX1	0.032	0.054	0.062	0.019	−0.303	−0.309	−0.336	−0.304
COX2	0.154	0.163	0.142	0.105	−0.369	−0.375	−0.350	−0.350
COX3	**0.000**	**0.022**	**0.104**	**0.058**	−0.353	−0.367	−0.388	−0.398
ATP6	0.072	0.102	0.114	0.079	−0.569	−0.576	−0.599	−0.590
ATP8	0.186	0.209	0.283	0.196	−0.646	−0.707	−0.789	−0.775
Cytb	0.047	0.074	0.102	0.106	−0.463	−0.455	−0.458	−0.488
12S rRNA	0.206	0.202	0.244	0.195	−0.157	−0.343	−0.176	−0.147
16S rRNA	0.238	0.241	0.280	0.243	−0.170	−0.181	−0.209	−0.203
CR	**−0.169**	**−0.182**	**0.027**	**/**	−0.194	−0.141	−0.379	/
13PCG	0.054	0.066	0.087	0.067	−0.402	−0.414	−0.425	−0.422
overall	0.092	0.098	0.180	0.133	−0.375	−0.382	−0.396	−0.416

Note: The bold values indicated significant differences between the 4 species.

### 3.2 Protein‐coding genes and codon usage patterns

The 13 mitochondrial PCGs of *G. himalayensis* were 11,393 bp in length, which accounted for 65.54% of the total mitogenome sequence. The PCG regions of the *G. himalayensis* mitogenome consisted of 3,797 codons in total. Most of the PCGs had ATG as start codon, except for COXI and ND6, which had GTG and ATT start codons, respectively. This is the case in the other *Gyps* species as well (Table 1; Jiang et al., 2015; Liu et al., 2017). As in the case of mitogenomes of other vertebrates, TAA was the most frequent stop codon in *G. himalayensis*. ND2 had incomplete stop codon consisting of T‐, COX I, and ND1 ending with AGG and ending with TAG. These have been presumably added by post‐transcriptional polyadenylation using poly‐A tail (Ojala, Montoya, & Attardi, 1981). All other stop codons had the standard terminal codon (TAA). Among the 64 available codons, the most commonly used ones were isoleucine (L; AUU; 7.14%), phenylalanine (F; UUU; 4.99%), leucine (L; CUU; 4.49%), and leucine (L; CUA; 3.82%). The most frequently used amino acid in *G. himalayensis* was isoleucine (Table 3). In contrast, the codons ACG, CCG, UCG, CGG, AAG, GCG, and CAG were rarely used, accounting for only 1.38% of the amino acids. Moreover, just like in other vertebrates (Li, Liu, et al., 2015; Li, Huang, et al., 2015; Zhang, Nie, Wang, & Hu, 2009), a strong bias against G at the third codon position was found in all the 13 PCGs. The base composition of three codon positions of the 13 PCGs is shown in Table S2. The A + T content of the first (56.37%), the second (56.12%), and the third positions (56.41%). Hence, the third position has the highest A + T content, whereas the second position has the highest C + G content (43.88%; first position: 43.63%; third position: 43.59%). Moreover, the first positions have a positive AT‐ and negative GC‐skews, but the second and third positions have negative AT‐ and GC‐skews (Table S2). Among the 13 PCGs in the *G. himalayensis* mitogenome (Table 1), the ND3 has the highest (56.1%) and the ND6 the lowest A + T content (50.1%).

**Table 3:** Codon usage in *Gyps himalayensis* mitochondrial protein‐coding genes

Codon	Count	RSCU	%	Codon	Count	RSCU	%	Codon	Count	RSCU	%	Codon	Count	RSCU	%
UUU(F)	188	1.41	4.99	UCU(S)	59	1.22	1.57	UAU(Y)	59	1.13	1.57	UGU(C)	17	1.26	0.45
UUC(F)	79	0.59	2.1	UCC(S)	41	0.85	1.09	UAC(Y)	45	0.87	1.2	UGC(C)	10	0.74	0.27
UUA(L)	137	1.40	3.64	UCA(S)	137	2.83	3.64	UAA(*)	3	1.71	0.08	UGA(W)	95	1.76	2.52
UUG(L)	14	0.14	0.37	UCG(S)	8	0.17	0.21	UAG(*)	1	0.57	0.03	UGG(W)	13	0.24	0.35
CUU(L)	169	1.73	4.49	CCU(P)	20	0.40	0.53	CAU(H)	27	0.57	0.72	CGU(R)	13	0.70	0.35
CUC(L)	92	0.94	2.44	CCC(P)	54	1.08	1.43	CAC(H)	68	1.43	1.81	CGC(R)	19	1.03	0.5
CUA(L)	144	1.47	3.82	CCA(P)	117	2.34	3.11	CAA(Q)	79	1.80	2.1	CGA(R)	38	2.05	1.01
CUG(L)	31	0.32	0.82	CCG(P)	9	0.18	0.24	CAG(Q)	9	0.20	0.24	CGG(R)	4	0.22	0.11
AUU(I)	269	1.53	7.14	ACU(T)	69	0.95	1.83	AAU(N)	64	1.01	1.7	AGU(S)	21	0.43	0.56
AUC(I)	83	0.47	2.2	ACC(T)	86	1.19	2.28	AAC(N)	63	0.99	1.67	AGC(S)	24	0.50	0.64
AUA(M)	139	1.56	3.69	ACA(T)	127	1.75	3.37	AAA(K)	74	1.80	1.97	AGA(*)	1	0.57	0.03
AUG(M)	39	0.44	1.04	ACG(T)	8	0.11	0.21	AAG(K)	8	0.20	0.21	AGG(*)	2	1.14	0.05
GUU(V)	63	1.39	1.67	GCU(A)	73	0.91	1.94	GAU(D)	29	0.77	0.77	GGU(G)	45	0.79	1.2
GUC(V)	37	0.82	0.98	GCC(A)	147	1.84	3.9	GAC(D)	46	1.23	1.22	GGC(G)	69	1.21	1.83
GUA(V)	63	1.39	1.67	GCA(A)	94	1.18	2.5	GAA(E)	64	1.52	1.7	GGA(G)	73	1.28	1.94
GUG(V)	18	0.40	0.48	GCG(A)	6	0.07	0.16	GAG(E)	20	0.48	0.53	GGG(G)	42	0.73	1.12

### 3.3 Transfer and ribosomal RNA genes

**Figure 4:**
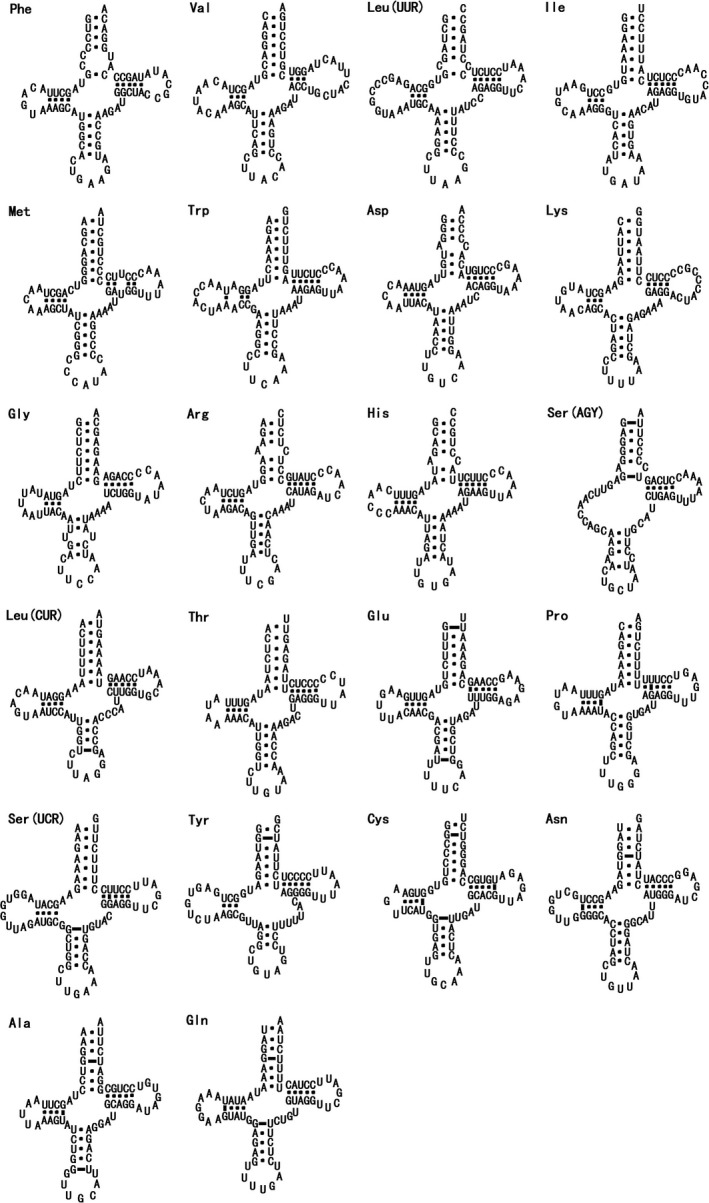
Putative tRNA secondary structures predicted from the 22 tRNA gene sequences found in the *Gyps himalayensis* mitogenome

In total, the typical 22 tRNA genes with conventional secondary structures were found from the mitogenome of *G. himalayensis*. The total length of mitochondrial tRNA genes was 1,548 bp and size of individual genes ranged from 65 bp (tRNA‐Ser2) to 74 bp (tRNA‐Leu1 and tRNA‐Leu2). Most of the tRNAs could be folded into the canonical cloverleaf secondary structure, while the tRNA‐Ser (S1) contains a predicted secondary structure with the TΨC arm and loop, but lacks the DHU arm and loop. This phenomenon is considered to be a typical feature of vertebrate mitogenomes (Lu, Lu, Li, & Jiang, 2016; Wolstenholme, 1992). Moreover, most of anticodons were identical to those observed in other *Gyps* species. The CCA 3′‐terminal group was added during processing. A total of 24 unmatched base pairs were found in the tRNAs of *G. himalayensis*, and these contained G–U pairs in the 9 bp AA, 6 bp A–C, 5 bp DHU and 4 bp TψC stems (Figure 4). Stem mismatches appear to be ordinary in tRNA genes and possibly repaired through a post‐transcriptional editing process (Lavrov, Brown, & Boore, 2000). Compared to other birds, most mismatched nucleotides were G‐U pairs, which can form weak bonds in tRNAs and noncanonical pairs in tRNA secondary structures (Gutell, Lee, & Cannone, 2002; Li, Liu, et al., 2015; Li, Liu, et al., 2015).

The ribosomal genes (12S rRNA and 16S rRNA) were identified as to location, length, and base composition in the mitogenome of the *G. himalayensis*. The 12S rRNA gene is situated between tRNA‐Phe and tRNA‐Val, while the 16S rRNA gene is located between tRNA‐Val and tRNA‐Leu (Figure 3). The length of 12S rRNA and 16S RNA were 985 bp and 1,630bp, respectively (Table 1). The base composition of the 12S rRNA was 20.2%T, 28.4%C, 30.7%A, and 20.7%G, with an A + T content of 50.9%, while the 16S ribosomal gene composition was 20.4%T, 27.2%C, 33.1%A, and 19.3%G, with an A + T content of 53.4%. The gene order and arrangement of two rRNA genes, including gene length, base composition, and RNA structure, were similar to those in other Accipitridae birds (Jiang et al., 2015; Li, Liu, et al., 2015; Li, Liu, et al., 2015; Song et al., 2015).

### 3.4 Noncoding regions

The noncoding region of the *G. himalayensis* mitogenome comprised of two major control regions. First one is the short noncoding region (pseudo‐control region, CCR) 619bp in length, which is located after tRNA‐Glu gene. The second one (CR) is 1,202 bp long and located between tRNA‐Thr and tRNA‐Pro (Figure 3). The CCR sequence is shortest than any other sequences in the mitogenome (Table 4). The CRs of both vertebrates and invertebrates have a high A + T content and the replication initiation feature (Boore, 1999). The base frequencies in the CR of *G. himalayensis* are 33.1% T, 25.9% C, 23.5% A, and 17.5% G, while those of the pseudo‐control region (CCR) are 35.2% T, 21.5% C, 33.9% A, and 9.4% G, respectively.

**Table 4:** Sequence characteristics of pseudo‐control regions (CCRs) in Falconiformes birds

Family	Genus	Scientific name	Accession number	CCR			
				length (bp)	Types	Tandem repeats	Single repeat unit
Accipitridae	*Gyps*	*Gyps hinalayensis*	KY594709	689	2	12.8 × (11)	5′‐TCTTTTTTCAT
						6.9 × (44)	3'‐CCCTAAACAAGTAATAATATAAGTAGATGAGCTATCTACAAAGC
Accipitridae	*Aegypius*	*Aegypius monachus*	KF682364	1,025	5	2.0 × (20)	5'‐TAATCTTTCTTTCCACTTTT
						25.1 × (11)	5'‐CACTTTATTTT
						12.2 × (43)	5'‐CAAGTCTACAAGCCCTAAACAAGTAATTATATAAGCAGATGAG
						3.1 × (176)	3'‐CAAGTCTACAAGCCCTAAACAAGTAATTATATAAGCAAGGAGCAAGTATACAAGCCCTAAACAAGTAAATATATATTAGCAGGGCGAACGTGTACACAACCCTACACAAGTATATATATAAGCAGATGAGCAAGTCTACAAGCCCTAAACAAGTAATTATATAAGCAGATGAG
						2.4 × (219)	3'‐CAAGTCTACAAGCCCTAAACAAGTAATTATATACTAGCAGGCGAACAAGTACACAAGCCCTAAACAAATAATTATATACAGCAGGCGAACAAGTACACAAGCCCTAAACAAGTATATATATAAGCAGATGAGCAAGTCTACAAGCCCTAAACAAGTAATTATATAAGCAGATGAGCAAGTCTACAAGCCCTAAACAAGTAATTATATAAGCACAGGAG
Accipitridae	*Spilornis*	*Spilornis cheela*	NC_015887	1532	3	7.6 × (10)	5'‐TATGTATATA
						4.2 × (16)	5'‐TATATACATGTATATA
						8.3 × (48)	3'‐AACAGATGAGCAATTCTACAAAAGCCATGAAACAAATGACTAACGATT
Accipitridae	*Spizaetus*	*Spizaetus alboniger*	AP008239	1,273	1	17.5 × (46)	3'‐TAACCAACAATATAATGAATGATACGTTCTATAAACCGTCAATCAA
Accipitridae	*Accipiter*	*Accipiter virgatus*	KJ699124	1,092	4	3.5 × (11)	5'‐CATCTTTATTA
						2.6 × (49)	5'‐ATAACTAGTAACCCCGGCGGCAGTGCATAATTTCAAAATCATGAAATAT
						14.9 × (48)	3'‐AAGCCGTGAAATAGCAACTACTATATAAAAGAATGAGCAGTCTTATAG
						3.2 × (25)	5'‐AGCAGTCTTATAGAAGCCGTGAAAT
Accipitridae	*Hieraaetus*	*Hieraaetus fasciatus*	KP329567	1799	1	18.4 × (49)	3'‐CCAACAATATAAATGAATGATTAGCGCTAATAAAGCCGTGAGCAAGTAA
Accipitridae	*Buteo*	*Buteo buteo*	AF380305	1,455	1	23.2 × (48)	5'‐AGCAATACTACCAAAGCCCTAAACAAGTAACTAATACTCTAAGCAATG
Accipitridae	*Aquila*	*Aquila chrysaetos*	KF905228	627	1	2.3 × (49)	3'‐AACCAACAATATAAATGAATGATTAGTTCTAATAGAGCCGTGAACAAAT
Falconidae	*Falco*	*Falco peregrinus*	AF090338	950	2	28.9 × (27)	3'‐ATAAACGAAATAGGTGAG
						3.7 × (18)	3'‐ATAAACGAAATAGGTGAG
Falconidae	*Falco*	*Falco tinnunculus*	NC_011307	796	3	36.2 × (18)	3'‐AACAAAACAAACAAAATA
						3.5 × (19)	5'‐CAAACAAAACAACCAAAATTAAA
						65.2 × (9)	3'‐TAAACGAAA
Falconidae	*Micrastur*	*Micrastur gilvicollis*	NC_008548	1539	1	6.4 × (69)	3'‐CATCACTTTTTATCATGACAACAACTAAAATTAACTTAAACTCCCCTACCTAACACAATCAAACTTTTTT
Pandionidae	*Pandion*	*Pandion haliaetus*	KF961184	**–**	**–**	**–**	
Sagittariidae	*Sagittarius*	*Sagittarius serpentarius*	NC_008550	**–**	**–**	**–**	

Note

CCR = pseudo‐control region; 7 (48) =7 repeats of 48 bp (single repeat unit is 48‐bp in length); 3'‐ = the repetitive region close to 3' region of CCR.

**Figure 5:**
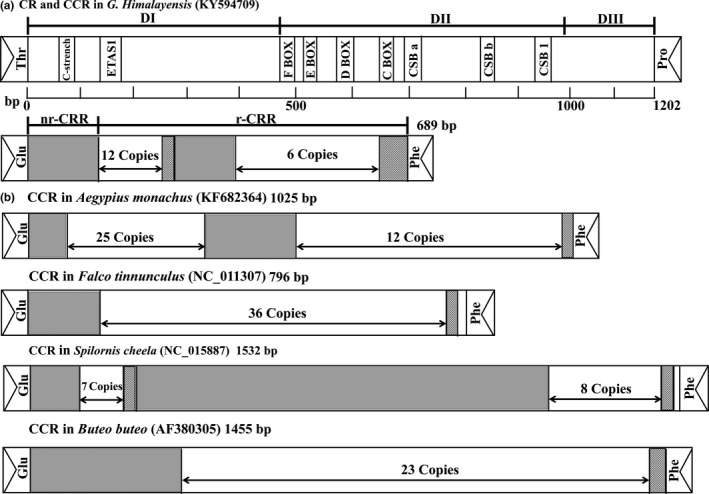
(a) Structure of CR and CCR in *Gyps himalayensis*. Positions of the conserved motifs in CR and the division into the three domains DI, DII and DIII are shown. (b) Structure of CCR in *Aegypius monachus*, *Spilornis cheela*, *Buteo buteo,* and *Falco tinnunculus*. nr‐CCRs are depicted as gray bars. Regions containing incomplete units are depicted as hatched bars. CCR, pseudo‐control region; nr‐CCR, nonrepetitive region in CCR; r‐CCR, repetitive region in CCR

*Gyps himalayensis* control region has the same structure as in *Aegypius monachus* (Cadahíaet al., 2009; Li, Liu, et al., 2015; Li, Liu, et al., 2015), and it possesses common characteristics for other birds (Roques, Godoy, Negro, & Hiraldo, 2004; Ruokonen & Kvist, 2002). In general, three distinct CR domains are recognized: (a) the highly variable, left‐end domain I; (b) the conserved, central domain (DII); (c) and the right‐end domain III (DIII); Baker & Marshall, 1997). These three distinct domains were present also in the griffon vulture CR (Figure 5a).

**Figure 6:**
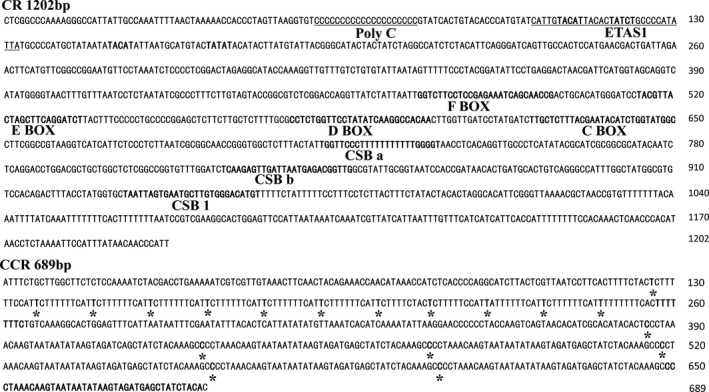
Nucleotide sequence of the mitochondrial control region (CR) and pseudo‐control region (CCR) of *Gyps himalayensis*. Several conserved motifs were identified along this fragment: putative ETAS1 elements (underlined), palindromic motifs (TACAT, ATGTA), boxes (F, E, D, C), blocks (CSB a, CSB b, CSB1), and repeated sequences (* and bold initials). In complete repeated units are depicted in bold

At the 5′‐end of the DI, we observed a long continuous poly‐C sequence (Figure 6). This structure seems to be a conserved feature, present also in Accipitridae, Sagittariidae, Falconidae, and Pandionidae mitogenomes (Cadahía et al., 2009; Haring et al., 2001). Conserved 5′‐TACAT‐3′ and 5′‐ATGTA‐3′ palindromic motifs (Saccone, Pesole, & Sbisa,^1991^) were observed at the 5′ end of *G. himalayensis* CR (Figure 6). The four conserved (C, D, E, and F) boxes positioned in the central domain and the CSB a, CSB b, and CSB1 regions were also found in *G. himalayensis* (Figures 5a and 6). However, we failed to locate the conserved blocks CSB2 and CSB3, speculated to be involved in the initiation of DNA synthesis (Sbisa, Tanzariello, Reyes, Pesole, & Saccone, 1997). Similarly, these boxes (ETAS2, CSB2, and CSB3) have been reported to be absent in other birds such as *Accipiter virgatus* (Song et al., 2015), *Ciconia ciconia* (Yamamoto et al., 2000), and *B. buteo* (Haring et al., 2001; Figure 5b).

As shown in Figure 5b, the pseudo‐control region of *G. himalayensis* had a 125 bp nonrepeating region (nr‐CCR) in the 5′ end, followed by a cluster of tandem repeats in the 3′ end (r‐CCR): 12 times of 11 bp repeats units (followed by a 8 bp incomplete repeat unit), then 120 bp insertion and more than six times of 44 bp repeats units (followed by a 40 bp incomplete repeat unit; Figure 5, Table 4). The type and number of repeat units in CCR is very variable in Falconiformes (Table 4), and we could not find any similarity between the 11 bp and the 44 bp repeat units. Similarly, we observed two clusters of repetitive regions in the CCR of *A. monachus* (Figure 5b): the first one (11 bp per unit, 25 times) at the 5′ region, and the second one (43 bp per unit, 12 times) at the 3′ region. Interestingly, these two regions are neither close to each other (with a 148 bp nr‐CCR in between them), nor showed any similarity to each other. Nonrepetitive regions of raptor CCR have been observed in most birds, for example, 118 bp in *S. cheela* (Qin et al., 2013), 169 bp in *F. peregrinus* (Mindell, Sorenson, & Dimcheff, 1998), 338 bp in *B. buteo* (Haring, Riesing, Pinsker, & Gamauf, 1999), 360 bp in *Haliaeetus albicilla* (Haring et al., 2001), and 472 bp in *Aquila chrysaetos* (Masuda et al., 1998). None of them had recognizable repeats from tRNA‐Glu or ND6 genes, as was observed in *Amazona* parrots (Eberhard, Wright, & Bermingham, 2001). These variable tandem repeats have been identified as the main reason for the length variability of mitochondrial genome control regions, as well as for the whole mitogenome (Kim, Seong, Ho, & Ha, 1998; Zhang et al., 2016).

### 3.5 Phylogenetic relationships

**Figure 7:**
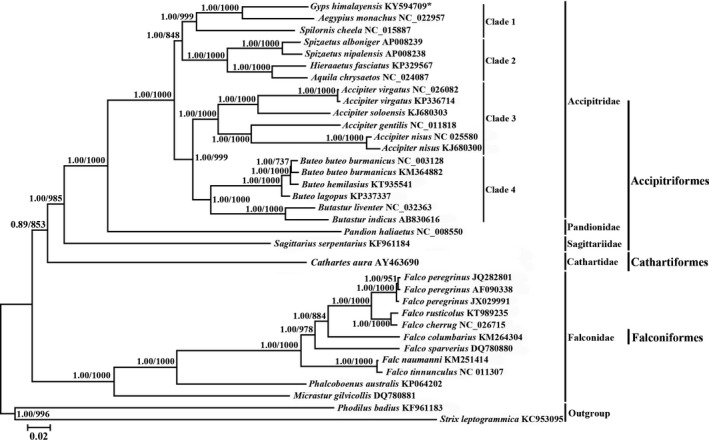
Results of phylogenetic analyses based on Bayesian inference (BI) and maximum likelihood (ML) analyses for 32 raptor taxa based on 13 PCGs sequences. *Phodilus badius* (KF961183) and *Strix leptogrammica* (KC953095) were used as outgroups. Tree topologies produced by BI and ML analyses were identical. Bayesian posterior probability and bootstrap support values from ML analyses, respectively, are shown on the nodes. The asterisks indicate new sequence generated in this study

The nucleotide data for phylogenetic analyses included 11,364 nucleotide sites from 13 protein‐encoding genes. Of these sites, 5,472 were conservative, 5,889 were variable, and 4,835 sites were parsimoniously informative. So far, based on a total of 33 other complete mitogenome sequences retrieved from NCBI GenBank (Table S1), BI and ML trees showed identical topology, and most internal nodes were well supported by posterior probabilities and bootstrap values, respectively (Figure 7). The results indicate that mitochondrial genome sequences of 33 Raptors could be divided into three branches: Accipitriformes, Cathartiformes, and Falconiformes (Figure 7). Accipitriformes and Cathartiformes are sister branches, which are then grouped with Falconiformes. Accipitriformes includes three subclades: Accipitridae, Pandionidae, and Sagittariidae. The monophyly of Accipitridae was strongly supported (Figure 7). New World vultures (Cathartidae) have some similar characters in common with storks, and a close phylogenetic relationship with storks was proposed (Seibold & Helbig, 1995; Sibley & Monroe, 1990; Wink, 1995). However, recent molecular studies have shown that the New World vultures clearly have their affinity with many raptors and not with storks (Burleigh et al., 2015; Hackett et al., 2008; Wink & Sauer‐Gürth, 2004). Our phylogeny indicated that *C. aura* (Cathartidae) was basal to the remaining Accipitriformes, which is consistent with Jiang et al.’s results (Jiang et al., 2015). According to a relatively short sequence, Wink and Sauer‐Gürth (2004) placed *S. serpentarius* with storks. In our phylogenetic analysis, the *S. serpentarius* is deepest on the branch with *Pandion haliaetus* (Pandionidae) and Accipitridae (Figure 7). This result is consistent with some previous studies (Hackett et al., 2008; Lerner & Mindell, 2005; Mahmood et al., 2014). Furthermore, *P. haliaetus* is the sister to Accipitridae. The relationships among the four families of Accipitriformes are consistent with Burleigh et al.’s results (Burleigh et al., 2015; Jiang et al., 2015). In addition, the phylogenetic results indicate that there were four clades in Accipitridae. Clade 1 was (*Gys* + *Aegypius* + Spilornis), Clade 2 was (*Aquila* + *Hieraaetus* + Spizaetus), Clade 3 was only *Accipiter,* and the Clade 4 was (*Buteo* + *Butastur*) (Figure 7).

Our results indicated that the relationships at the family level were (Falconidae + (Cathartidae + (Sagittariidae + (Accipitridae + Pandionidae))), and this inference was supported by both BI (posterior probabilities ≥0.89) and ML (bootstrap ≥85.3%) analyses (Figure 7). The Falconidae clade contained members from the genera *Falco*, *Phalcoboenus,* and *Micrastur*, and all the *Falco* species formed a monophyletic cluster (Figure 7). *Falco* and *Phalcoboenus* species were resolved as the sister group of the clade in genus *Micrastur* (1.00, 100%). In agreement with traditional taxonomy (Mahmood et al., 2014; Sibley, Ahlquist, & Monroe, 1988; Wang et al., 2016), *F. tinnunculus* and *F. naumanni* formed the earliest diverging lineage within the *Falco*. The Accipitridae included the genera *Gyps*, *Aegypius*, *Spilornis*, *Spizaetus*, *Aquila*, *Hieraaetus*, *Butastur*, *Accipiter*, *Buteo*, and *Spizaetus*. In this clade, *S. cheela* was a taxon ancestral to *G. himalayensis* and *A. monachus*. Furthermore, the results of the phylogenetic analyses support a closer relationship between *G. himalayensis* and *A. monachus*, rather than between *G. himalayensis* and *S. cheela* (Figure 7). This is in accordance with the results of Jiang et al. (2014) and Wink (1995). *P. haliaetus* forms a sister group with the family Accipitridae clade. Clade Sagittaridae comprised of *S. serpentarius* as the sister taxon to the Accipitridae and Pandionidae (1.00, 98.5%). Also, this result was consistent with those of the previous studies (Lerner, Klaver, & Mindell, 2008; Liu et al., 2017; Song et al., 2015), but differed from those of Hackett et al. (2008) and Pacheco et al. (2011). The difference is mainly due to the different samples used and molecular markers, and their evolutionary relationships need further investigating and searching for more evidences from binding nuclear gene data and morphological characters. In the current study, all clades were well resolved, with only a few exceptions less than 80%, while Bayesian posterior probabilities were 1.00 (except for one node). Despite their fast evolutionary rates, mitogenomes contain species‐specific evolutionary relationships, which can be efficiently recovered by improving taxon sampling (Rubinstein et al., 2013).

## 4 CONCLUSIONS

In short, this is the first study to report and analyze the complete mitogenome of the *G. himalayensis*. The sequence structure of the *G. himalayensis* mitogenome is typical for birds and possesses high similarity to other reported Accipitridae mitogenomes. However, at the 5′ end of the DI region, a long continuous poly‐C sequence was discovered. Additionally, two types of tandem repeat units were also found in the pseudo‐control region. The phylogenetic analyses support a closer relationship between *G. himalayensis* and *A. monachus* than that between *G. himalayensis* and *S. cheela*. Consequently, the results should provide a useful resource for future study of *Gyps* mitochondrial genomic evolution. However, the proposed evolutionary relationships among *G. himalayensis*, *A. monachus*, and *S. cheela* based on the findings that emerged in the current study should be accepted with caution due to limited taxon sampling. Many aspects of the phylogeny of the genus *Gyps* remain to be resolved and further analysis based on more molecular markers (binding nuclear gene) and a large number of taxon sampling is necessary to clarify the phylogenetic relationships among species of genus *Gyps*, *Aegypius*, and *Spilornis*.

## CONFLICT OF INTEREST

The authors declare that there are no conflicts of interest.

## AUTHOR CONTRIBUTIONS

Jiang L and Ruan Q conceived and designed the experiments, performed the experiments, analyzed the data. Peng L, Tang M, and Zhang M performed field work and laboratory work. You Z and Zhang M analyzed the data. Jiang L, West A, Merilä J, and Chen W participated in writing the manuscript.

## Data Availability

The following information was supplied regarding the deposition of DNA sequences: GenBank accession numbers: KY594709.
