# Supplementary material for: Complete mitochondrial genome sequence of the Himalayan Griffon, Gyps himalayensis (Accipitriformes: Accipitridae): Sequence, structure, and phylogenetic analyses
Source: Ecol Evol. 2019 Jul 9;9(15):8813–28. doi: 10.1002/ece3.5433 (PMC6686361; doi:10.1002/ece3.5433)
Supplement: Supplementary file 1 [file ECE3-9-8813-s001.docx]

**TABLE S1** Comparison of whole genome sizes and A + T contents, together with GenBank accession numbers of the mitogenomes of 32 Falconiformes birds.

| **Species** | **Accession no.** | **Total** | | | | **13 PCGs** | | | | **16S rRNA gene** | | | | **12S rRNA gene** | | | | **22 tRNA** | | | | **CR** | | | |
| --- | --- | --- | --- | --- | --- | --- | --- | --- | --- | --- | --- | --- | --- | --- | --- | --- | --- | --- | --- | --- | --- | --- | --- | --- | --- |
|  |  | **Length (bp)** | **A+T (%)** | **AT skew** | **GC skew** | **Length (bp)** | **A+T (%)** | **AT skew** | **GC skew** | **Length (bp)** | **A+T (%)** | **AT skew** | **GC skew** | **Length (bp)** | **A+T (%)** | **AT skew** | **GC skew** | **Length (bp)** | **A+T (%)** | **AT skew** | **GC skew** | **Length (bp)** | **A+T (%)** | **AT skew** | **GC skew** |
| *Gyps himalayensis* | KY594709 | 17381 | 54.04 | 0.092 | -0.375 | 10610 | 52.80 | 0.054 | -0.402 | 1630 | 53.44 | 0.238 | -0.17 | 985 | 50.86 | 0.206 | -0.157 | 1548 | 57.30 | 0.134 | -0.189 | 1890 | 61.16 | -0.107 | -0.251 |
| *Nisaetus nipalensis* | NC_007598 | 17667 | 53.39 | 0.118 | -0.396 | 11393 | 51.80 | 0.063 | -0.425 | 1597 | 52.85 | 0.213 | -0.193 | 969 | 48.92 | 0.224 | -0.168 | 1357 | 57.26 | 0.133 | -0.2 | 1158 | 57.25 | -0.161 | -0.200 |
| *Buteo buteo* | AF380305 | 18674 | 55.15 | 0.128 | -0.413 | 11396 | 52.97 | 0.076 | -0.444 | 1598 | 55.01 | 0.245 | -0.191 | 972 | 51.65 | 0.183 | -0.187 | 1547 | 56.95 | 0.156 | -0.213 | 1672 | 63.28 | -0.191 | -0.186 |
| *Buteo buteo burmanicus* | KM364882 | 18231 | 55.01 | 0.120 | -0.414 | 11393 | 52.95 | 0.076 | -0.446 | 1593 | 54.99 | 0.251 | -0.197 | 971 | 51.18 | 0.179 | -0.177 | 1547 | 57.01 | 0.154 | -0.212 | 1675 | 64.54 | -0.190 | -0.182 |
| *Buteo hemilasius* | KT935541 | 18079 | 54.84 | 0.118 | -0.415 | 11394 | 52.80 | 0.080 | -0.449 | 1593 | 55.12 | 0.248 | -0.189 | 972 | 51.65 | 0.187 | -0.191 | 1547 | 56.88 | 0.152 | -0.208 | 230 | 75.65 | -0.034 | -0.857 |
| *Buteo lagopus* | KP337337 | 18559 | 54.95 | 0.131 | -0.417 | 11394 | 52.70 | 0.082 | -0.448 | 1594 | 55.02 | 0.245 | -0.191 | 973 | 51.59 | 0.179 | -0.185 | 1546 | 56.99 | 0.156 | -0.212 | 1654 | 63.66 | -0.181 | -0.178 |
| *Hieraaetus fasciatus* | KP329567 | 18513 | 54.20 | 0.123 | -0.390 | 11394 | 53.02 | 0.062 | -0.432 | 1594 | 53.51 | 0.215 | -0.193 | 971 | 50.15 | 0.211 | -0.161 | 1550 | 56.65 | 0.137 | -0.188 | 1159 | 57.20 | -0.158 | -0.198 |
| *Accipiter gentilis* | NC_011818 | 18266 | 55.89 | 0.139 | -0.424 | 11392 | 54.12 | 0.085 | -0.441 | 1595 | 56.36 | 0.230 | -0.155 | 971 | 52.32 | 0.205 | -0.184 | 1541 | 57.56 | 0.150 | -0.208 | 1977 | 61.81 | -0.020 | -0.335 |
| *Accipiter soloensis* | KJ680303 | 17900 | 55.75 | 0.071 | -0.418 | 11391 | 54.46 | 0.079 | -0.449 | 1597 | 56.11 | 0.234 | -0.207 | 969 | 51.39 | 0.229 | -0.206 | 1549 | 57.46 | 0.155 | -0.217 | 1276 | 58.54 | -0.183 | -0.142 |
| *Accipiter nisus* | KJ680300 | 19417 | 57.65 | 0.113 | -0.418 | 11392 | 55.11 | 0.061 | -0.425 | 1599 | 55.60 | 0.213 | -0.169 | 972 | 52.16 | 0.211 | -0.183 | 1549 | 57.65 | 0.140 | -0.204 | 2289 | 64.92 | -0.117 | -0.260 |
| *Accipiter nisus* | NC_025580 | 18647 | 56.96 | 0.100 | -0.406 | 11395 | 55.00 | 0.062 | -0.425 | 1602 | 55.37 | 0.211 | -0.183 | 971 | 52.11 | 0.213 | -0.183 | 1548 | 57.56 | 0.143 | -0.205 | 2329 | 65.01 | -0.125 | -0.261 |
| *Accipiter virgatus* | KP336714 | 17654 | 54.75 | 0.130 | -0.407 | 11395 | 53.54 | 0.089 | -0.447 | 1600 | 55.38 | 0.239 | -0.207 | 972 | 51.95 | 0.228 | -0.199 | 1548 | 57.04 | 0.148 | -0.203 | 1243 | 58.00 | -0.184 | -0.207 |
| *Accipiter virgatus* | NC_026082 | 17952 | 54.95 | 0.130 | -0.401 | 11391 | 53.55 | 0.088 | -0.446 | 1600 | 55.38 | 0.242 | -0.207 | 972 | 51.65 | 0.219 | -0.204 | 1548 | 57.11 | 0.149 | -0.205 | 1308 | 58.10 | -0.195 | -0.219 |
| *Aegypius monachus* | NC_022957 | 17811 | 54.03 | 0.098 | -0.382 | 11398 | 52.44 | 0.066 | -0.414 | 1628 | 53.93 | 0.241 | -0.181 | 981 | 51.07 | 0.202 | -0.167 | 1553 | 56.73 | 0.140 | -0.208 | 1225 | 55.92 | -0.182 | -0.141 |
| *Pandion haliaetus* | DQ780884 | 17864 | 55.05 | 0.121 | -0.385 | 11400 | 54.75 | 0.091 | -0.417 | 1581 | 54.59 | 0.270 | -0.181 | 971 | 51.91 | 0.246 | -0.173 | 1542 | 57.13 | 0.153 | -0.189 | 1156 | 56.06 | -0.173 | -0.256 |
| *Spilornis cheela* | JN191388 | 18291 | 53.02 | 0.144 | -0.409 | 11394 | 51.42 | 0.086 | -0.425 | 1598 | 53.50 | 0.242 | -0.176 | 972 | 49.69 | 0.230 | -0.182 | 1549 | 55.71 | 0.147 | -0.207 | 1144 | 55.86 | -0.155 | -0.224 |
| *Aquila chrysaetos* | KF905228 | 17332 | 53.33 | 0.115 | -0.399 | 11386 | 52.38 | 0.064 | -0.426 | 1537 | 54.33 | 0.200 | -0.16 | 968 | 49.17 | 0.227 | -0.163 | 1553 | 56.99 | 0.141 | -0.189 | / | / | / | / |
| *Spizaetus alboniger* | NC_007599 | 17977 | 53.47 | 0.125 | -0.400 | 11393 | 51.68 | 0.069 | -0.427 | 1589 | 52.67 | 0.216 | -0.184 | 969 | 49.33 | 0.218 | -0.157 | 1543 | 56.90 | 0.148 | -0.209 | 1162 | 57.31 | -0.168 | -0.177 |
| *Butastur liventer* | NC_032363 | 19673 | 56.34 | 0.107 | -0.375 | 11396 | 53.04 | 0.059 | -0.426 | 1602 | 55.06 | 0.247 | -0.197 | 972 | 52.26 | 0.189 | -0.164 | 1547 | 56.50 | 0.146 | -0.201 | 1516 | 61.08 | -0.302 | -0.234 |
| *Butastur indicus* | NC_032362 | 19059 | 55.80 | 0.101 | -0.397 | 11396 | 52.95 | 0.055 | -0.422 | 1602 | 55.68 | 0.231 | -0.18 | 972 | 51.65 | 0.207 | -0.174 | 1550 | 56.58 | 0.133 | -0.186 | 1543 | 61.24 | -0.306 | -0.261 |
| *Falco peregrinus* | AF090338 | 18068 | 55.54 | 0.180 | -0.389 | 11402 | 54.28 | 0.086 | -0.423 | 1599 | 53.72 | 0.281 | -0.208 | 979 | 52.20 | 0.245 | -0.171 | 1563 | 56.69 | 0.160 | -0.205 | / | / | / | / |
| *Falco peregrinus* | JX029991 | 17527 | 54.91 | 0.163 | -0.403 | 11401 | 54.23 | 0.085 | -0.422 | 1599 | 53.85 | 0.280 | -0.209 | 979 | 52.40 | 0.244 | -0.172 | 1583 | 56.22 | 0.166 | -0.227 | / | / | / | / |
| *Falco peregrinus* | JQ282801 | 18115 | 55.56 | 0.180 | -0.396 | 11401 | 54.25 | 0.087 | -0.425 | 1599 | 53.85 | 0.280 | -0.209 | 979 | 52.40 | 0.244 | -0.176 | 1569 | 56.60 | 0.160 | -0.207 | 1635 | 57.43 | 0.027 | -0.379 |
| *Falco cherrug* | KP337902 | 18059 | 55.68 | 0.176 | -0.389 | 11401 | 54.28 | 0.081 | -0.422 | 1599 | 53.78 | 0.272 | -0.196 | 982 | 51.43 | 0.240 | -0.174 | 1562 | 57.11 | 0.159 | -0.197 | / | / | / | / |
| *Falco columbarius* | NC_025579 | 17516 | 54.38 | 0.179 | -0.420 | 11395 | 53.41 | 0.092 | -0.427 | 1595 | 54.61 | 0.288 | -0.202 | 979 | 51.17 | 0.277 | -0.192 | 1566 | 57.22 | 0.163 | -0.221 | 1414 | 55.37 | 0.022 | -0.448 |
| *Falco sparverius* | DQ780880 | 17507 | 54.49 | 0.163 | -0.409 | 11394 | 53.89 | 0.086 | -0.423 | 1599 | 54.72 | 0.275 | -0.213 | 983 | 50.05 | 0.297 | -0.206 | 1566 | 56.90 | 0.156 | -0.206 | / | / | / | / |
| *Falco tinnunculus* | EU196361 | 17663 | 54.39 | 0.200 | -0.417 | 11395 | 53.19 | 0.106 | -0.435 | 1596 | 53.95 | 0.280 | -0.219 | 978 | 49.08 | 0.288 | -0.189 | 1568 | 56.63 | 0.180 | -0.224 | 1266 | 54.50 | -0.006 | -0.361 |
| *Falco naumanni* | KM251414 | 17370 | 53.80 | 0.187 | -0.421 | 11395 | 53.05 | 0.107 | -0.434 | 1600 | 54.00 | 0.282 | -0.215 | 916 | 48.91 | 0.295 | -0.184 | 1566 | 56.64 | 0.188 | -0.222 | 1337 | 54.15 | 0.008 | -0.374 |
| *Falco rusticolus* | KT989235 | 18218 | 55.50 | 0.178 | -0.394 | 11401 | 54.14 | 0.085 | -0.425 | 1599 | 53.85 | 0.278 | -0.203 | 984 | 51.42 | 0.245 | -0.176 | 1570 | 57.07 | 0.163 | -0.214 | 1673 | 57.38 | -0.010 | -0.374 |
| *Phalcoboenus australis* | KP064202 | 17074 | 52.71 | 0.156 | -0.415 | 11384 | 52.40 | 0.091 | -0.438 | 1601 | 51.84 | 0.292 | -0.222 | 974 | 50.00 | 0.298 | -0.211 | 1554 | 55.66 | 0.163 | -0.225 | / | / | / | / |
| *Micrastur gilvicollis* | DQ780881 | 17344 | 54.47 | 0.133 | -0.416 | 11389 | 53.61 | 0.067 | -0.422 | 1610 | 54.97 | 0.243 | -0.203 | 974 | 53.08 | 0.195 | -0.147 | 1548 | 55.81 | 0.148 | -0.219 | / | / | / | / |
| *Sagittarius serpentarius* | KF961184 | 16773 | 54.56 | 0.125 | -0.411 | 11415 | 54.36 | 0.068 | -0.427 | 1610 | 53.60 | 0.231 | -0.205 | 972 | 51.75 | 0.233 | -0.198 | 1560 | 56.92 | 0.158 | -0.223 | 1117 | 58.01 | -0.025 | -0.258 |
| *Cathartes aura* | AY463690 | 16779 | 54.28 | 0.123 | -0.375 | 11397 | 53.49 | 0.057 | -0.394 | 1606 | 54.55 | 0.237 | -0.164 | 973 | 48.41 | 0.205 | -0.161 | 1539 | 57.63 | 0.029 | -0.034 | 1177 | 58.71 | 0.100 | -0.329 |

**TABLE S2** Base composition and AT/CG skews for the 13 PCGs in *Gyps himalayensis* mitogenome.

| **Species** | **A(%)** | **T(%)** | **C(%)** | **G(%)** | **A+T(%)** | **C+G(%)** | **AT-skew** | **GC-skew** |
| --- | --- | --- | --- | --- | --- | --- | --- | --- |
| *Gyps hinalayensis* | 25.73 | 30.56 | 28.83 | 14.86 | 56.30 | 43.70 | -0.086 | -0.320 |
| 1st | 28.54 | 27.82 | 29.33 | 14.29 | 56.37 | 43.63 | 0.013 | -0.345 |
| 2nd | 25.31 | 30.79 | 26.85 | 17.02 | 56.12 | 43.88 | -0.098 | -0.224 |
| 3th | 23.33 | 33.07 | 30.32 | 13.26 | 56.41 | 43.59 | -0.173 | -0.391 |
